# Supplementary material for: MetaQTL: a package of new computational methods for the meta-analysis of QTL mapping experiments
Source: BMC Bioinformatics. 2007 Feb 8;8:49. doi: 10.1186/1471-2105-8-49 (PMC1808479; doi:10.1186/1471-2105-8-49)
Supplement: Additional File 7 — MetaQTL Package : jar file and tutorial. This Zip archive contains both the MetaQTL JAR file and the files of the tutorial. [file 1471-2105-8-49-S7.zip › org.inra.metaqtl/doc/org/metaqtl/graph/package-summary.html]

org.metaqtl.graph


|  |  |  |
| --- | --- | --- |
| **Overview** \| **Package** \| Class \| **Use** \| **Tree** \| **Deprecated** \| **Index** \| **Help** | |  |
| **PREV PACKAGE**   **NEXT PACKAGE** | **FRAMES**    **NO FRAMES**     **All Classes** |  |


---

## Package org.metaqtl.graph

| **Class Summary** | |
| --- | --- |
| **AnchoredElement** |  |
| **ChromAxe** |  |
| **ChromGraph** | A `ChromGraph` allows to link together a `Chromosome` and a `QtlPartition` in order to be depicted into a graphic. |
| **ChromLayer** |  |
| **ChromShape** |  |
| **GraphFactory** |  |
| **GraphShape** |  |
| **Layer** |  |
| **MetaGraph** |  |
| **MetaGraphPar** |  |
| **PaletteColor** |  |
| **QtlLayer** |  |
| **QtlLayerBuffer** |  |
| **QtlPartitionLayer** |  |
| **QtlShape** |  |
| **QtlTreeLayer** |  |
| **QtlUnit** |  |

---


|  |  |  |
| --- | --- | --- |
| **Overview** \| **Package** \| Class \| **Use** \| **Tree** \| **Deprecated** \| **Index** \| **Help** | |  |
| **PREV PACKAGE**   **NEXT PACKAGE** | **FRAMES**    **NO FRAMES**     **All Classes** |  |


---
